# Medical Society of the County of Erie

**Published:** 1901-08

**Authors:** Franklin C. Gram

**Affiliations:** Secretary


### SOCIETY PROCEEDINGS.

### Medical Society of the County of Erie.

*Eightieth Semiannual Meeting.*

Reported by FRANKLIN C. GRAM, M. D., Secretary.

THE president, Wm. C. Phelps, called the 80th semiannual meeting of the Medical Society of the County of Erie to order at 10.30 a.m., June 11, 1901, in the parlors of the Young Men's Christian Association.

The secretary read the minutes of the previous meeting, which were adopted.

Dr. Wm. Warren Potter, of the committee on membership, presented the names of the following applicants, and recommended their election: Drs. J. Henry Dowd, Samuel H. Lynde, Geo. H. McMichael and Theodore V. Bauer. The recommendation was adopted by the society and all were declared elected.

Dr. Chas. E. Congdon, of the special committee, appointed at the previous meeting, asked for more time for his committee, which was granted.

Dr. Wm. Warren Potter called attention to the recent change in the by-laws of the State Society, whereby the representation from each county society was increased to five delegates from each assembly district under similar conditions as formerly, without increasing the cost to the society. This would increase the delegation from Erie County to the state society to forty, instead of eight, as heretofore. There are usually a number of applicants for the honor, but as it is desirable to elect only such as will qualify for permanent membership it is advisable to canvass before election. He therefore moved that a committee, consisting of the officers of the society and the chairman of the membership committee, be appointed to present names of members to be elected as such delegates.

The resolution was adopted and later this committee presented the following names of members who were elected to serve for three years with the delegates elected in January, 1901: Drs. Wm. C. Phelps, E. H. Ballou, R. S. Myers, F. E. Fronczak, Julius Ullman, John H. Grant, Wm. F. May, C. E. Congdon, D. H. Sherman, C. P. Smith, G. R. Trowbridge, G. F. Cott, F. E. L. Brecht, J. J. Mooney, Joseph Fowler, C. A. Wall, Julius Pohlman, B. G. Long, W. M. Ward, J. H. Dowd, J. B. Coakley, Henry R. Lapp and A. E. Woehnert.

Chairman J. B. Coakley, of the Board of Censors, submitted the following report, which was received and filed:

*To the Medical Society of the County of Erie:*

Your Board of Censors respectfully reports that since its last report, made and received at the meeting of this society on the 9th day of January last, the censors have endeavored to perform faithfully the work of investigating cases of the illegal practice of medicine and surgery. Complaints have been received against several individuals and these complaints have been carefully investigated. The persons to whom they refer have been visited and in two or three cases our investigation and visit has resulted in the discontinuance of the illegal practises complained of, which, in most instances have seemed to be the result of ignorance and mistake rather than of deliberate intention to violate the law.

In our last report we made reference to the case of one Arthur de Collard, and stated that we had under consideration the advisability of further prosecuting his case. While this matter was under consideration Mr. de Collard found it advisable to depart from the city, and, as we understand from this state, and therefore such proceedings became unnecessary.

As noted also in our last report, our counsel during the last session of the legislature endeavored to have the medical law amended in several important particulars, referred to in our last report, and to that end had conferences with the representatives of the Medical Society of the State of New York, and the Medical Society of the County of New York, at Albany, during the session of the legislature. He reports that he found that the proposed act to amend the medical law had been so drawn as to contain provisions especially aimed at the practice of so-called christian science, osteopathy and the like, and that very early in the session he became convinced that the drastic and far reaching character of the proposed amendments was such that it did not seem probable that the legislature would adopt them. As you have no doubt learned from the public press, there were a number of hearings before the legislative committees in regard to these amendments, which were embodied in what was known as the Bell Medical Bill, and the final result was, as anticipated, that the legislature declined to pass these amendments and that the whole subject of medical legislation which was involved with them was put on the shelf until another year. Our counsel did not participate in these hearings on one side or the other, as he did not believe it good policy to do so under the circumstances. We hope and believe that at the next session of the legislature the practical and sensible amendments which we have suggested and advocated will receive favorable consideration at the hands of the legislature.

Respectfully submitted.

J. B. Coakley, Chairman. Henry C. Lapp.
H. R. Hopkins, Francis E. Fronczak,
Irving W. Potter.

Dr. P. W. VanPeyma called attention to the death of Dr. Nichell, one of the oldest and most respected members of this society, which had not come to the notice of the officers in time to call a special meeting to take the usual action, and he therefore presented the following memorial which was unanimously adopted:

IN MEMORIAM—HENRY NICHELL, M. D.

Dr. Henry Nichell was born at Mainz, on the Rhine, July 17, 1821. He graduated from the University of Giessen in 1847. The following year he married Charlotte Erkell Kuhn, who at the time of her marriage to Dr. Nichell was a widow with two small children. These children, a boy and a girl, were adopted by the doctor and treated with paternal affection. The son, Dr. Charles Nichell, died some years ago in San Francisco, California. The daughter is now Mrs. Minna J. Roese, of 27 Day's Park, Buffalo, N.Y.

Dr. Nichell came to Buffalo September 17, 1848. For a short time he practised on Main Street, but soon settled at No. 80 Sycamore Street, where he continued his practice for a period of about forty-eight years. He graduated a second time from the University of Buffalo in 1857. He early joined the Medical Society of the County of Erie and he was for many years, and until the time of his death, a member of the American Medical Association. He died February 14, 1901, at the home of his adopted daughter.

Dr. Nichell was rather short of stature and slight of figure, neat, quiet, and gentlemanly in appearance. In the care of his patients he was kind, painstaking and conscientious. He was a man of the strictest integrity and possessed of a high ethical sense; extremely modest and yet very proud to be a member of the medical profession. He was a diligent student and his knowledge of medicine was far above the average, and so recognised by his professional associates. Pathology and materia medica were perhaps his favorite studies. He had paid special attention to the diseases of children, and the size of his practice attested for many years the general appreciation of knowledge and skill. As he was proud of his profession, so the medical profession has reason to be proud of him—a modest, conscientious, intelligent worker in the ranks.

The secretary stated that the parlors of the Y.M.C.A. had been placed at the gratuitous disposal of the society, and recommended that a gift of $5.00 be granted the janitor for his extra work. The recommendation was adopted.

The president then introduced Dr. Henry L. Elsner, of Syracuse, President of the Medical Society of the State of New York, who had honored the society with his presence, and who, after paying several compliments to the Erie County Society, read a carefully prepared paper on The diagnosis of malignant endocarditis.

Dr. Suiter, of Herkimer, a former president of the state society, was likewise presented and briefly acknowledged the compliment.

Dr. Elsner's paper was discussed by Drs. Suiter, Hopkins, Clark, E. H. Long and P. W. VanPeyma.

Dr. Henry R. Hopkins read a paper on The fan system of heating, ventilating and air supply of certain Buffalo schools. (See page 1.)

Prof. H. M. Hill presented a paper on Ideal school ventilation, but owing to the lateness of the hour Dr. T. B. Carpenter's paper on School ventilation as it exists, was read by title.

Prof. Henry P. Emerson, superintendent of public schools discussed these papers.

A vote of thanks was tendered Dr. Elsner for his interesting paper, after which the society adjourned.

